# Early Six-Month Functional and Radiological Outcomes of Expert Tibia Nailing in Intra-articular Tibial Plateau Fractures: A Prospective Study

**DOI:** 10.7759/cureus.111560

**Published:** 2026-06-26

**Authors:** Vishal Kumar, Jayant Kothale, Ravi Sharma, Mohamed P Azlam

**Affiliations:** 1 Orthopaedics, Netaji Subhas Medical College and Hospital, Bihta, Patna, IND; 2 Orthopaedics, Government Medical College, Seoni, IND; 3 Orthopaedics, Netaji Subhash Chandra Bose Medical College, Jabalpur, IND

**Keywords:** expert tibia nail, intramedullary nailing of the tibia, minimally invasive surgery, schatzker’s classification, tibial plateau fractures

## Abstract

Background and objective

Intra-articular tibial plateau fractures present significant management challenges. Traditional plating can compromise soft tissues, whereas Expert Tibia Nailing provides minimally invasive fixation with multidirectional locking capabilities. This study aimed to evaluate the early functional and radiological outcomes of Expert Tibia Nailing, with an a priori comparison between simple (Schatzker I-IV) and complex (V-VI) tibial plateau fractures.

Methods

This prospective observational study included 36 patients treated with Expert Tibia Nailing at the Department of Orthopaedics, Netaji Subhash Chandra Bose Medical College, Jabalpur, from May 2022 to June 2024. The cohort comprised 29 males (80.6%) and seven females (19.4%), with a mean age of 41.6 ± 11.5 years. The primary outcome was the Knee Society Score (KSS) at six months. Secondary outcomes included time to weight-bearing, radiological union, and complications. Categorical variables were analyzed using the chi-square test or Fisher’s exact test, and continuous variables were analyzed using the Mann-Whitney U test.

Results

Road traffic accidents accounted for injuries in 27/36 patients (75.0%), while falls accounted for 9/36 cases (25.0%). Schatzker type II, type IV, and type V fractures were each observed in 7/36 patients (19.4%). The mean time to partial weight-bearing was 8.11 ± 1.8 weeks, and the mean time to radiological union was 15.83 ± 2.9 weeks. At six months, KSS outcomes were rated as excellent in 10/36 patients (27.8%), good in 16/36 (44.4%), fair in 4/36 (11.1%), and poor in 6/36 (16.7%). Excellent-to-good outcomes were achieved in 26/36 patients (72.2%). Patients with Schatzker types I-IV demonstrated significantly better outcomes than those with types V-VI (20/23, 87.0% vs. 6/13, 46.2%; p = 0.007). Complications occurred in 14/36 patients (38.9%), including knee stiffness in 7/36 (19.4%), condylar collapse in 6/36 (16.7%), superficial infection in 3/36 (8.3%), and malunion in 1/36 (2.8%). No cases of nonunion, deep infection, compartment syndrome, implant failure, or neurovascular injury were observed.

Conclusions

Expert Tibia Nailing yielded satisfactory early functional and radiological outcomes in this prospective series of tibial plateau fractures, with low rates of deep infection, nonunion, and neurovascular complications. However, the technique demonstrated poorer results and a higher complication burden in bicondylar fractures; therefore, its use should be limited to carefully selected unicondylar patterns. Larger multicenter studies with longer follow-up periods are needed to confirm its role relative to plate-based fixation.

## Introduction

Tibial plateau fractures account for 1-2% of long bone fractures and pose substantial challenges due to their intra-articular nature and high complication rates [1,2]. Management principles emphasize anatomical restoration, stable fixation, early mobilization, and soft tissue preservation [3]. Traditional open reduction and internal fixation (ORIF) allows direct visualization of the fracture but can compromise soft tissues, thereby increasing the risks of infection and wound-related complications [4,5].

Intramedullary nailing has emerged as an attractive alternative, offering biological fixation with minimal soft tissue disruption, load-sharing biomechanics, and preservation of fracture hematoma [6]. The Expert Tibia Nailing System, introduced in 2005, incorporates unique design features including multidirectional locking options in proximal and distal segments, angular stability, and a more distal Herzog bend to prevent anterior angulation of proximal fragments [7]. These modifications enable treatment of metaphyseal and intra-articular fractures previously considered unsuitable for intramedullary fixation.

Recent literature suggests favorable outcomes with intramedullary nailing for selected tibial plateau fractures, with some studies reporting equivalent or superior results compared to plate fixation regarding union time, functional scores, and complication rates [8,9]. However, comprehensive data evaluating Expert Tibia Nailing across all Schatzker fracture types remains limited, particularly concerning intra-articular involvement. This study aims to evaluate early functional and radiological outcomes of Expert Tibia Nailing, with an a priori comparison between simple (Schatzker I-IV) and complex (V-VI) tibial plateau fractures, providing evidence-based insights for clinical decision-making.

## Materials and methods

Study design and setting

This prospective observational study was conducted at the Department of Orthopaedics, Netaji Subhash Chandra Bose Medical College, Jabalpur, India, from May 2022 to June 2024. Institutional Ethics Committee approval was obtained (approval letter no. IEC/NSCBMC/22/21-24), and all participants provided written informed consent. Eligible consecutive patients presenting during the study period were included in the analysis, and no formal a priori sample size or power calculation was performed because this was a prospective single-center observational series.

Patient selection

Inclusion criteria included adults aged 25-60 years with closed or Gustilo-Anderson type I-II open tibial plateau fractures of any Schatzker classification, presenting within four weeks of injury, and willing to comply with follow-up. Exclusion criteria included pathological fractures, Gustilo-Anderson type III open fractures, concomitant neurovascular injuries requiring repair, ipsilateral lower limb fractures, active infection, medical contraindications to surgery, and refusal to participate.

Three patients with Gustilo-Anderson type I-II open fractures were included in the study because these low-grade open injuries were considered amenable to early definitive fixation after adequate debridement. An additional reason for their inclusion was that these low-grade open injuries were managed with the same standardized protocol of urgent irrigation, debridement, and definitive fixation used for the rest of the cohort. Although this introduced a small degree of heterogeneity, low-grade open fractures were considered suitable for internal fixation after adequate soft-tissue management and were therefore not excluded. No routine bone graft or bone substitute was used in any case because the operative strategy in this series was to evaluate Expert Tibia Nailing as a minimally invasive fixation method rather than to replicate plate osteosynthesis protocols.

Preoperative assessment

Patients underwent comprehensive clinical evaluation and standard radiographs. CT with 3D reconstruction was performed for complex patterns. Fractures were classified using Schatzker and AO/OTA (Arbeitsgemeinschaft für Osteosynthesefragen/Orthopaedic Trauma Association) systems. Preoperative templating determined nail dimensions. All open fractures were managed with urgent operative irrigation and meticulous surgical debridement of the wound and contaminated tissue, followed by assessment of fracture stability and soft-tissue viability. Broad-spectrum intravenous antibiotics were administered preoperatively, and tetanus prophylaxis was provided as indicated. Definitive fixation was performed after satisfactory debridement, depending on soft-tissue condition and wound status.

Surgical technique

​​​​​​Patients were positioned supine with the knee flexed 90 degrees. Surgery was performed under spinal or general anesthesia with image intensifier guidance. The infrapatellar approach was used for nail insertion, with the limb positioned under fluoroscopic control. The entry point was established at the anterior third-posterior two-thirds junction and confirmed in AP and lateral views. Depressed fragments were elevated percutaneously using Steinmann pins/elevators, and temporary fixation was achieved with Kirschner wires before nail insertion. Proximal locking was performed with multidirectional screws placed in a planned subchondral rafting configuration to support the articular fragments, and distal interlocking was performed with transverse screws under fluoroscopic guidance. Preoperative CT with 3D reconstruction was selectively obtained in fractures with suspected depression, comminution, bicondylar involvement, or unclear morphology on plain radiographs. All procedures were performed by trained orthopaedic trauma surgeons with prior experience in the Expert Tibia Nailing system, and non-blinded clinicians performed Knee Society Score (KSS) assessments.

Postoperative management

Immediate postoperative care included limb elevation, prophylactic antibiotics (cefuroxime 1.5g IV eight-hourly for 48 hours then oral for five days), and chemical thromboprophylaxis. Rehabilitation commenced with static quadriceps exercises and ankle pumps. Passive knee range-of-motion (0-60 degrees) began immediately with non-weight-bearing for weeks zero to four. Progressive active-assisted exercises (0-90 degrees) with partial weight-bearing (25-50% body weight) were performed during weeks four to eight. Full weight-bearing progressed from week eight onwards based on radiological callus formation.

Follow-up and outcomes

Clinical and radiological assessments were conducted at four weeks, eight weeks, three months, and six months. The primary outcome was functional recovery using KSS: excellent (≥80), good (70-79), fair (60-69), poor (<60) [10]. Secondary outcomes included time to weight-bearing, radiological union (bridging callus on three cortices), and complications (infection, malunion, nonunion, knee stiffness, implant failure). The KSS (© by The Knee Society) was used in accordance with The Knee Society’s conditions of use. The KSS at six months was selected as an early functional outcome measure to capture short-term recovery after fixation and rehabilitation. This time point was chosen in keeping with prior tibial plateau fracture series that have reported early functional and radiological outcomes within a similar follow-up window, while recognizing that it does not capture late degenerative change.

Statistical analysis

Data were analyzed using SPSS Statistics version 25.0 (IBM Corp., Armonk, NY). Continuous variables were expressed as mean ± standard deviation (SD) and compared using the Mann-Whitney U test for non-normally distributed data. Categorical variables were presented as frequencies and percentages, analyzed using the chi-square test or Fisher's exact test. Statistical significance was set at p < 0.05.

## Results

Demographics and injury characteristics

Thirty-six patients completed six months of follow-up. There were 29 males (80.6%) and seven females (19.4%), with a mean age of 41.6 ± 11.5 years. The age distribution was as follows: seven patients (19.4%) were aged 25-30 years, 12 (33.3%) aged 31-40 years, 10 (27.8%) aged 41-50 years, and seven (19.4%) aged 51-60 years. Road traffic accidents accounted for 27/36 injuries (75.0%), whereas falls accounted for 9/36 injuries (25.0%). Right-sided fractures occurred in 21/36 patients (58.3%) and left-sided fractures in 15/36 (41.7%). Three of 36 fractures (8.3%) were Gustilo-Anderson type I open injuries, and the remaining 33 (91.7%) were closed injuries.

Fracture pattern distribution

Based on the Schatzker classification, type I fractures were identified in 5/36 patients (13.9%), type II in 7/36 (19.4%), type III in 4/36 (11.1%), type IV in 7/36 (19.4%), type V in 7/36 (19.4%), and type VI in 6/36 (16.7%). According to the AO/OTA classification, fracture types 41B1, 41B2, and 41B3 were observed in 10/36 (27.8%), 15/36 (41.7%), and 11/36 patients (30.6%), respectively. The most common fracture pattern was 41B2, reflecting typical lateral plateau injury mechanisms from combined valgus and axial loading forces.

Operative parameters and timeline

The mean operative time was 82.4 ± 16.7 minutes, with a mean intraoperative blood loss of 142.8 ± 45.3 mL. Partial weight-bearing was initiated at a mean of 8.11 ± 1.8 weeks; 26/36 patients (72.2%) commenced between six and eight weeks, 7/36 (19.4%) between 9 and 10 weeks, and 3/36 (8.3%) after 10 weeks. The mean time to achieve full weight-bearing was 14.6 ± 2.4 weeks. Radiological fracture union was achieved at a mean of 15.83 ± 2.9 weeks; 20/36 patients (55.6%) demonstrated union by 14 weeks, 11/36 (30.6%) between 15 and 18 weeks, and 5/36 (13.9%) between 19 and 26 weeks (Table 1).

**Table 1:** Fracture pattern distribution among study subjects

Fracture pattern	Frequency	Percentage (%)
Schatzker classification		
Type I (lateral split)	5	13.9
Type II (split-depression)	7	19.4
Type III (pure depression)	4	11.1
Type IV (medial plateau)	7	19.4
Type V (bicondylar)	7	19.4
Type VI (metaphyseal)	6	16.7
AO classification		
41B1 (split)	10	27.8
41B2 (split-depression)	15	41.6
41B3 (pure depression)	11	30.6

Functional outcomes

At six months, KSS outcomes were excellent in 10/36 patients (27.8%), good in 16/36 (44.4%), fair in 4/36 (11.1%), and poor in 6/36 (16.7%). The mean KSS was 74.8 ± 12.6. Satisfactory outcomes, defined as excellent or good, were achieved in 26/36 patients (72.2%). The mean final knee flexion was 118.3 ± 18.7 degrees. A full range of motion was achieved in 17/36 patients (47.2%), a partial range of motion in 14/36 (38.9%), and persistent stiffness with less than 90 degrees of flexion was observed in 5/36 (13.9%) (Table 2).

**Table 2:** Descriptive analysis of functional and radiological outcomes

Outcome	Frequency	Percentage
Knee Society Score		
Excellent (≥80)	10	27.7%
Good (70-79)	16	44.4%
Fair (60-69)	4	11.1%
Poor (<60)	6	16.6%
Weight-bearing time		
6-8 weeks	26	72.2%
9-10 weeks	7	19.4%
>10 weeks	3	8.3%
Union time		
≤14 weeks	20	55.6%
15-18 weeks	11	30.6%
19-26 weeks	5	13.8%

Exploratory analysis of open versus closed fractures

An exploratory descriptive comparison was performed between the three Gustilo-Anderson type I open fractures and the closed fracture group. The open-fracture subgroup showed union and functional recovery that were broadly comparable with those of the closed-fracture group, although the small number of open injuries precluded formal statistical comparison. No deep infection occurred in the open-fracture subgroup, and the overall functional outcomes in these patients were comparable to those observed in the cohort as a whole. Because of the very small sample size, these findings should be interpreted as hypothesis-generating only.

Complications

Postoperative complications were observed in 14 of 36 patients (38.9%). Knee stiffness occurred in seven patients (19.4%), condylar collapse in six patients (16.7%), superficial infection in three patients (8.3%), and malunion in one patient (2.8%). Complications were concentrated in complex fractures: Schatzker V-VI injuries accounted for most cases of condylar collapse and the highest overall complication burden, whereas uncomplicated healing and satisfactory functional recovery were more frequent in Schatzker I-IV fractures. No nonunion, deep infection, compartment syndrome, implant failure, or neurovascular injury was observed.

Fracture type-specific analysis

Patients with Schatzker type I-IV fractures had significantly better functional outcomes than those with type V-VI fractures. Excellent-to-good KSS outcomes were observed in 20/23 patients (87.0%) with types I-IV and in 6/13 patients (46.2%) with types V-VI (p = 0.007). Mean union time was shorter in simple fracture patterns than in complex fracture patterns (14.2 ± 2.1 weeks vs. 18.9 ± 3.4 weeks; p < 0.001). Complications were observed in 4/23 patients (17.4%) with simple patterns and 10/13 patients (76.9%) with complex patterns (p < 0.001). Condylar collapse occurred only in complex fractures, affecting 6/13 patients (46.2%) (Table 3).

**Table 3:** Comparative outcomes between simple and complex fracture patterns ^*^Statistically significant KSS: Knee Society Score; SD: standard deviation

Parameter		Simple (I-IV), n = 23	Complex (V-VI), n = 13	Test of significance
KSS outcomes, n (%)	Excellent/good	20 (86.9%)	6 (46.2%)	χ² = 7.32, p = 0.007^*^
	Fair/poor	3 (13.1%)	7 (53.8%)	
Union time, mean ± SDS		14.2 ± 2.1 weeks	18.9 ± 3.4 weeks	t = 5.12, p < 0.001^*^
Complications, n (%)		4 (17.4%)	10 (76.9%)	χ² = 13.87, p < 0.001^*^
Condylar collapse, n (%)		0 (0%)	6 (46.2%)	χ² = 14.56, p < 0.001^*^

In the simple fracture group, complications were infrequent, whereas the complex fracture group showed a markedly higher rate of adverse events, indicating that nail-only fixation was less reliable in bicondylar patterns.

## Discussion

The principal finding of this study extends beyond the observation that Expert Tibia Nailing achieved acceptable early outcomes in selected cases; rather, it demonstrates that clinical and radiological outcomes were strongly influenced by fracture pattern. While simple Schatzker I-IV fractures showed relatively better union and functional recovery, complex Schatzker V-VI fractures had a much higher complication burden, including condylar collapse and poorer KSS outcomes. This pattern suggests that nail-only fixation may be reasonable for carefully selected unicondylar injuries, but it is not a dependable stand-alone strategy for bicondylar plateau fractures, where maintenance of medial column support is critical.

These findings are less favorable than contemporary reports of bicondylar tibial plateau fixation using dual plating or hybrid nail-plate constructs. Studies of bicondylar fractures treated with dual plating have reported acceptable complication rates and more reliable restoration of articular alignment, while combined intramedullary nail and plate fixation has been advocated to improve stability in complex proximal tibial patterns. In contrast, the present nail-only series showed inferior control of reduction in complex fractures, indicating that stand-alone Expert Tibia Nailing should not be extrapolated to Schatzker V-VI injuries based on these data.

This prospective study evaluating Expert Tibia Nailing for intra-articular tibial plateau fractures demonstrates satisfactory functional outcomes in 26 (72.2%) of patients, with superior results in unicondylar versus bicondylar patterns. The demographic profile - young adult males (mean 41.6 years, 29/36 (80.6%) males) injured predominantly by road traffic accidents (27/36 (75.0%)) - aligns with established epidemiological patterns from developing nations [11,12]. The technique offers distinct biomechanical advantages through multidirectional proximal locking, providing angular stability in wide metaphyseal bone, and optimized Herzog bend preventing anterior fragment angulation [7,13]. Mean operative time (82.4 minutes) and blood loss (142.8 mL) compare favorably with open plating (120-150 minutes, 250-400 mL) [14], reflecting minimally invasive benefits.

Fracture-pattern analysis revealed markedly superior outcomes in Schatzker types I-IV (20/23 (86.9%) satisfactory) versus types V-VI (6/13 (46.2%), p = 0.007), with significantly shorter union times (14.2 vs. 18.9 weeks, p < 0.001). This underscores technical challenges of bicondylar management with intramedullary fixation, where maintenance of medial column stability remains problematic. Recent literature advocates hybrid fixation combining medial plating with nailing for bicondylar patterns [9]. The finding of 26/36 (72.2%) satisfactory outcomes in the present study is consistent with contemporary series reporting excellent-to-good outcomes ranging from 70% to 76.6% following expert nailing [15,16]. However, comparative studies show plate fixation achieving 85% satisfactory outcomes [17], though these often exclude severely comminuted patterns. A recent multicenter randomized trial found no significant functional differences between nailing and plating at one-year follow-up [8], suggesting equivalent efficacy in appropriately selected cases.

Radiological union at 15.83 weeks compares favorably with other expert nailing studies reporting 18-20 weeks [18,19], reflecting preserved periosteal blood supply and biological healing. Early partial weight-bearing at 8.11 weeks enabled mobilization crucial for preventing knee stiffness while providing mechanical stimulation for fracture healing [20]. However, this shorter union time should be interpreted cautiously, as it may be influenced by the small sample size, fracture-pattern distribution, and selection of cases in this single-center series. Therefore, the observed union time is best considered a descriptive finding from this cohort rather than evidence of superior biological healing.

The 14/36 (38.9%) complication rate warrants consideration. Knee stiffness (7/36 (19.4%)) reflects the inherent propensity of intra-articular fractures for adhesion formation. Condylar collapse (6/36 (16.7%)) occurred exclusively in bicondylar patterns, highlighting inadequate subchondral support in severely comminuted fractures. Adjunctive techniques including calcium phosphate cement augmentation or hybrid plate-nail constructs may enhance stability [21]. The absence of deep infections (0%) represents a significant advantage over plate fixation reporting 5-15% infection rates in high-risk scenarios.

This study has several limitations. It was a single-center prospective observational series with a modest sample size and heterogeneous Schatzker subgroups, which limited the strength of subgroup comparisons. There was no control group, and hence the results cannot be used to infer comparative effectiveness compared with plating or hybrid fixation. Follow-up was limited to six months, restricting interpretation to early outcomes and precluding assessment of late post-traumatic arthritis, delayed functional decline, and fixation-related failures that may emerge only with longer follow-up. In addition, KSS assessment was not blinded, introducing the possibility of observer bias, and preoperative CT was obtained selectively rather than uniformly, creating potential classification imprecision in more complex fractures. The very small number of open fractures also prevented meaningful comparison with closed injuries. Finally, the high complication and condylar collapse rates observed in Schatzker V-VI fractures substantially limit the generalizability of these findings to bicondylar fracture patterns, for which more stable fixation constructs may be preferable.

The inclusion of only three low-grade open fractures may have introduced minor heterogeneity, and their very small number precludes any meaningful subgroup inference. In addition, the absence of routine bone grafting reflects the study design and may confound direct comparison with plate-based tibial plateau fixation literature, in which bone grafting is commonly used to support depressed fragments or metaphyseal voids. These factors should therefore be interpreted as potential confounders when comparing our findings with results from plating studies.

Expert Tibia Nailing offers advantages in specific scenarios: polytrauma patients requiring damage control, compromised soft tissues precluding extensile approaches, and simple intra-articular fractures amenable to closed reduction. Complex bicondylar patterns may benefit from alternative strategies. Importantly, surgeon experience with suprapatellar techniques and articular reduction maneuvers plays a critical role in determining procedural success and outcomes.

## Conclusions

Expert Tibia Nailing demonstrated acceptable early (six-month) functional outcomes in a majority of patients with intra-articular tibial plateau fractures and supported timely radiological union, with relatively low incidences of deep infection, nonunion, and neurovascular complications. In contrast, bicondylar (Schatzker V-VI) fractures treated with this stand-alone technique showed a substantially higher overall complication burden and frequent condylar collapse, suggesting that Expert Tibia Nailing should be limited to carefully selected unicondylar (Schatzker I-IV) fracture patterns rather than being applied routinely in complex plateau injuries. These findings support Expert Tibia Nailing as a minimally invasive option when soft-tissue conditions preclude extensile approaches for less complex intra-articular fractures, whereas complex bicondylar fractures are likely better managed with fixation strategies that provide strong medial and lateral column stability, such as dual plating or combined nail-plate constructs. The present data do not support extending the indication for Expert Tibia Nailing to bicondylar tibial plateau fractures, given the increased complication burden and poorer functional outcomes observed in this subgroup. Larger multicentre studies with adequate power and long-term follow-up are required to define the comparative effectiveness, complication profile, and optimal indications of Expert Tibia Nailing relative to plate-based osteosynthesis across various tibial plateau fracture subtypes.

## References

[1] Wani IH, Ul Gani N, Yaseen M, Bashir A, Bhat MS, Farooq M (2017). Operative management of distal tibial extra-articular fractures - intramedullary nail versus minimally invasive percutaneous plate osteosynthesis. Ortop Traumatol Rehabil.

[2] Saxena A, Mahore SK, Gupta A, Sriwastava AK, Gupta M (2025). Comparative evaluation of functional outcomes following surgical and conservative management of distal tibia shaft fractures: a prospective observational study. J Orthop Case Rep.

[3] Wu DH, Zhang Y, Xu D, Lou WG, Zhang JY (2025). Evaluation of the effectiveness of suprapatellar versus infrapatellar approach in intramedullary nailing for the treatment of tibial fractures. Eur J Med Res.

[4] Teimouri M, Mirghaderi P, Parry JA, Ziaei A, Salimi M, Tahririan MA (2023). Intramedullary nail versus minimally invasive plate osteosynthesis for displaced extraarticular proximal tibia fractures: a prospective comparative cohort study. Eur J Orthop Surg Traumatol.

[5] Ren C, Li M, Sun L (2021). Comparison of intramedullary nailing fixation and percutaneous locked plating fixation for the treatment of proximal tibial fractures: a meta-analysis. J Orthop Surg (Hong Kong).

[6] Barei DP, Nork SE, Mills WJ, Henley MB, Benirschke SK (2004). Complications associated with internal fixation of high-energy bicondylar tibial plateau fractures utilizing a two-incision technique. J Orthop Trauma.

[7] Attal R, Hansen M, Kirjavainen M (2012). A multicentre case series of tibia fractures treated with the expert tibial nail (ETN). Arch Orthop Trauma Surg.

[8] Dunbar RP, Egol KA, Jones CB (2023). Locked plating versus nailing for proximal tibia fractures: a multicenter RCT. J Orthop Trauma.

[9] Raja RS, Ramaswamy AR, Sivamurugan S (2026). Suprapatellar nailing with condylar screw support in complex proximal tibia fractures: technical experience from a case series. J Orthop Case Rep.

[10] (2026). The Knee Society. The Knee Society Score. https://www.kneesociety.org/the-knee-society-score.

[11] Shahzad A, Ali HB, Shah SA, Sajid A, Ellapparadja P, Jamil W (2025). Analysis of proximal plating versus intramedullary nailing in the treatment of extra-articular proximal tibial fracture: a randomized prospective study. J Orthop Case Rep.

[12] Monahan KT, Zavras AG, Angelides GW, Altman GT, Altman DT, Westrick ER (2024). Extra-articular proximal tibia fracture fixation with locked plating versus intramedullary nailing: a meta-analysis. Injury.

[13] Hansen M, Mehler D, Hessmann MH, Blum J, Rommens PM (2007). Intramedullary stabilization of extraarticular proximal tibial fractures: a biomechanical comparison of intramedullary and extramedullary implants including a new proximal tibia nail (PTN). J Orthop Trauma.

[14] Prabhat V, Kujur A, Topno R, Kundu S, Gupta GK, Prasad VD (2025). A randomised trial comparing the outcome of expert tibia nailing and plating for distal tibial fractures. Ann Afr Med.

[15] Dragonas CG, Fontalis A, Iliadis A, Vris A, Mavrogenis A (2025). Intramedullary nailing versus plate fixation for the treatment of proximal tibia extra-articular fractures: a meta-analysis. Eur J Orthop Surg Traumatol.

[16] Franke J, Homeier A, Metz L (2018). Infrapatellar vs. suprapatellar approach to obtain an optimal insertion angle for intramedullary nailing of tibial fractures. Eur J Trauma Emerg Surg.

[17] Ozcan C, Turkmen I, Sokucu S (2020). Comparison of three different approaches for anterior knee pain after tibia intramedullary nailing. Eur J Trauma Emerg Surg.

[18] Elnewishy A, Elkholy M, Hamada A, Salem M (2023). Comparing minimally invasive percutaneous plate osteosynthesis with interlocking intramedullary nail fixation for the management of adult extra-articular distal tibial fractures: a comprehensive systematic review and meta-analysis. Cureus.

[19] Makaram NS, Sheppard J, Leow JM, Oliver WM, Keating JF (2024). Outcome following intramedullary nailing of tibial diaphyseal fractures: suprapatellar nail insertion results in superior radiographic parameters but no difference in mid-term function. J Bone Joint Surg Am.

[20] Egol KA, Tejwani NC, Capla EL, Wolinsky PL, Koval KJ (2005). Staged management of high-energy proximal tibia fractures (OTA types 41): the results of a prospective, standardized protocol. J Orthop Trauma.

[21] Welch RD, Zhang H, Bronson DG (2003). Experimental tibial plateau fractures augmented with calcium phosphate cement or autologous bone graft. J Bone Joint Surg Am.

